# Deep learning-based reconstruction of ultrasound images from raw channel data

**DOI:** 10.1007/s11548-020-02197-w

**Published:** 2020-06-03

**Authors:** Hannah Strohm, Sven Rothlübbers, Klaus Eickel, Matthias Günther

**Affiliations:** 1 Fraunhofer Institute for Digital Medicine MEVIS, Bremen, Germany; 2 University of Bremen, Bremen, Germany

**Keywords:** Plane wave ultrasound imaging, Deep learning

## Abstract

**Purpose:**

We investigate the feasibility of reconstructing ultrasound images directly from raw channel data using a deep learning network. Starting from the raw data, we present the network the full measurement information, allowing for a more generic reconstruction to form, as compared to common reconstructions constrained by physical models using fixed speed of sound assumptions.

**Methods:**

We propose a U-Net-like architecture for the given task. Additional layers with strided convolutions downsample the raw data. Hyperparameter optimization was used to find a suitable learning rate. We train and test our deep learning approach on plane wave ultrasound images with a single insonification angle. The dataset includes phantom as well as in vivo data.

**Results:**

The images produced by our method are visually comparable to ones reconstructed with the conventional delay and sum algorithm. Deviations between prediction and ground truth are likely to be related to speckle noise. For the test set, the mean absolute error is $4.23 \pm 1.52$ for the phantom images and $6.09 \pm 0.72$ for the in vivo data.

**Conclusion:**

The result shows the feasibility of our approach and opens up new research directions regarding information retrieval from raw channel data. As the networks reconstruction performance is limited by the quality of the ground truth images, using other ultrasound reconstruction technique or image types as target data would be of interest.

## Introduction

Recently, deep learning networks are explored as a replacement for ultrasound-related processing tasks like reconstruction, segmentation or compression. One important question arising when designing such networks is what kind of data representation to use as an input. Simson et al. [1] provided time-delayed scanline ultrasound data to a fully convolutional neural network and mapped them to ground truth images given by minimum variance beamforming. Beamformed data were used in [2] to learn a better compounding for plane wave imaging. Using raw radiofrequency channel data as input was proposed by Nair et al. [3, 4] for anechoic cyst segmentation.

Using already processed data instead of raw data has some advantages. First, dependent on the sampling rate, the raw data are often of a bigger size, which can cause memory issues while training networks. Second, processing steps like beamforming transform the raw data into a spatial domain where direct correspondence to the ultrasound image is given. On the other hand, all kinds of preprocessing steps work with constraints. For instance, the beamforming step in the popular delay and sum reconstruction algorithm assumes a constant speed of sound. This does not exactly represent the reality as most scanned tissues are composites.

In this short communication, we present our work on reconstructing nonoblique plane wave ultrasound images directly from unprocessed raw channel data using convolutional neural networks. By using the raw data instead of the beamformed data as the input, we give the network access to full measured information and the opportunity to learn a different way of beamforming. Similar approaches are pursued by Nair et al. [3, 4] but with a main focus on segmentation rather than on reconstruction. Furthermore, they used simulated raw data showing only one subject per frame, whereas we train and test our network on both diverse phantom and in vivo data.

## Methods

*Data* We acquired 2183 plane wave ultrasound images with a single parallel plane wave insonification using a DiPhAS ultrasound device (Fraunhofer Institute for Biomedical Engineering, St. Ingbert, Germany) with a linear 128-element transducer. Besides the images, which are reconstructed by the device with the delay and sum algorithm, also the respective raw data were recorded; 1281 images depict a phantom (Model 054GS, CIRS, Norfolk, USA) with acoustic scatterers of different sizes and reflectivities, and 902 show in vivo data of the abdominal area. The maximum penetration depth for all images was set to $92.4~\mathrm {mm}$, which corresponds to 4800 raw data samples given a sampling rate of $40~\mathrm {MHz}$. The pitch of $0.3~\mathrm {mm}$ between the single transducer elements defines the image width of $38.4~\mathrm {mm}$. The ultrasound images were of size $800~\mathrm {px}~\times ~256~\mathrm {px}$ with intensities in the range [0, 255].

As images were acquired in different sessions, not all of them display the whole depth of $92.4~\mathrm {mm}$. For those with a smaller depth, the pixel resolution was not changed but the raw data as well as the image data were filled up with zeros. We split the dataset randomly in $70\%$ training, $10\%$ validation and $20\%$ test data. Each subsplit contained phantom as well as in vivo data.

**Fig. 1:**
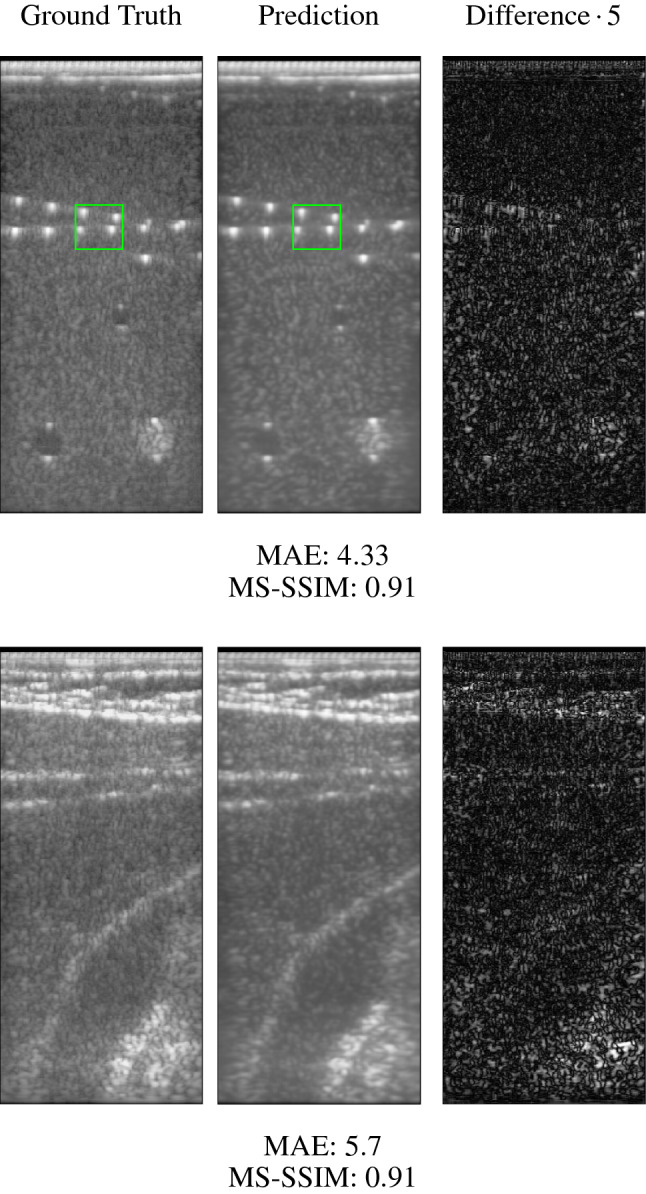
Qualitative comparison between ground truth computed with delay and sum and predicted reconstruction with our network. The examples are taken from the test set. The mean absolute error and the multiscale structural similarity index comparing the prediction to the ground truth are given. The difference images on the right display the absolute difference between ground truth and prediction (scaled by a factor of 5 for better visibility)

*Model* A four-level U-Net with some adaptions was utilized. Model definition was done with the Keras engine using the tensorflow backend. In order to handle the large difference in size between the raw data input and the image data output, two convolutional layers with strides 2 and 3 were added. Compared to [3], where the raw data were resampled to a smaller size, we hypothesize that the strided convolutions adapt to the downsampling task more efficiently and with lower loss of information. Five fully connected layers with decreasing numbers of neurons at the end of the network summarize the information in the different channels. In the downpath, LeakyReLu was used as activation function assuming that this will help the network to process the raw data input which can also be negative. We also used batch normalization before the activation layers and dropout with rate 0.1. As loss function, we used the ultrasound loss defined in [1], which is a combination of the peak signal-to-noise ratio and the multiscale structural similarity index (MS-SSIM). All training runs were performed with Adam as optimizer and batch size of 4, which was the maximum achievable size due to memory constraints.

In order to find a suitable learning rate for the network, we did hyperparameter optimization as described in [5], which combines Bayesian optimization and Hyperband. We sampled 15 different configurations with learning rates between $10^{-2}$ and $10^{-6}$ and evaluated them on different budgets according to the Hyperband scheme. A learning rate of $2.85~\times ~10^{-4}$ showed the best performance and was used for training the final network. We stopped the training after 20 epochs since no substantial performance gain either in the validation nor the training loss was visible.

**Fig. 2:**
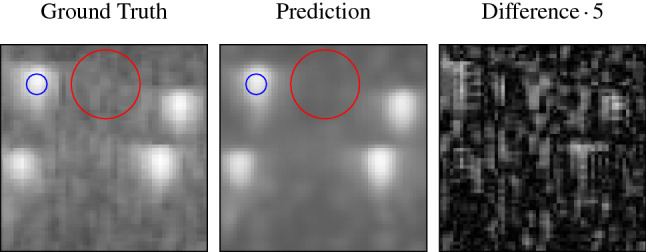
Enlarged sections that are marked in Fig. 1 by green boxes. The circles mark the regions that are used for an exemplary CNR computation (blue: signal, red: background)

## Results

Figure 1 displays a qualitative comparison of our networks reconstruction and the ground truth. Both predicted images, the phantom on the top and the in vivo image on the bottom, are visually comparable to the ground truth. The difference images between ground truth and prediction show only minor deviations, which are likely to be related to the speckle noise pattern, which is reduced in the predicted images. Loading the network takes around $1.92\hbox { s}$, while inference on the graphic card (Nvidia GeForce RTX 2070) needs on average $0.02\hbox { s}$ or approximately 50 frames per second.

An exemplary contrast-to-noise ratio (CNR) calculation following the definition in [6] was done, comparing the intensity of a phantom scatterer with the background. The respective regions are marked in Fig. 2 by colored circles. Here, the CNR for the prediction is with $19.27~\mathrm {dB}$ slightly better than for the ground truth ($17.94~\mathrm {dB}$).

For quantitative evaluation of the performance, Table 1 displays the mean and standard deviation of the mean absolute error (MAE) and the MS-SSIM for all images in the test set. The low values for the MAE and values of the MS-SSIM close to one support the qualitative impression of similarity of ground truth and prediction.

**Table 1:** Performance metrics evaluated on the test set

	Phantom	In vivo
MAE	$4.23 \pm 1.52$	$6.09 \pm 0.72$
MS-SSIM	$0.91 \pm 0.04$	$0.9 \pm 0.01$

## Conclusion

We introduced a neural network architecture reconstructing ultrasound images directly from the raw channel data. The results show the feasibility of this approach as the reconstruction from the network is of similar quality as the ground truth. One restriction of our approach is the quality of the target data: As the network is trained on images obtained with the delay and sum algorithm, it could hardly perform better than the reference reconstruction technique.

Therefore, for further investigations about the potential that lies in the full information content of the raw data, we would like to replace the target ultrasound image. Suitable candidates could be images showing other ultrasound contrasts or, in the case of plane wave imaging, are reconstructed using more insonification angles. Even images from other modalities like magnetic resonance imaging could be used.
